# Navigating conflicting value systems: a grounded theory of the process of public health equity work in the context of mental health promotion and prevention of harms of substance use

**DOI:** 10.1186/s12889-022-12627-w

**Published:** 2022-02-01

**Authors:** Lenora Marcellus, Bernie Pauly, Wanda Martin, Tina Revai, Kathy Easton, Marjorie MacDonald

**Affiliations:** 1 School of Nursing, University of Victoria, PO Box 1700 STN CSC, Victoria, BC V8W 2Y2 Canada; 2 School of Nursing and Scientist, Canadian Institute for Substance Use Research, University of Victoria, Box 1700 STN CSC, Victoria, BC V8W 2Y2 Canada; 3 College of Nursing, University of Saskatchewan, Health Science Building - 1A10, Box 6, 107 Wiggins Road, Saskatoon, SK S7N 5E5 Canada; 4 First Nations Health Authority, 501-100 Park Royal South Coast Salish Territory, West Vancouver, BC V7T-1A2 Canada; 5 Island Health, 345 Wale Rd, Victoria, BC V9B 6X2 Canada; 6 School of Nursing, and Scientist, Canadian Institute for Substance Use Research, University of Victoria, Box 1700 STN CSC, Victoria, Canada

**Keywords:** Grounded theory, health equity, public health, ethics, public health ethics, prevention of substance use harms, harm reduction, mental health promotion

## Abstract

**Background:**

Promoting health equity and reducing heath inequities is a foundational aim and ethical imperative in public health. There has been limited attention to and research on the ethical issues inherent in promoting health equity and reducing health inequities that public health practitioners experience in their work. The aim of the study was to explore how public health providers identified and navigated ethical issues and their management related to promoting health equity within services focused on mental health promotion and preventing harms of substance use.

**Methods:**

Semi-structured individual interviews and focus groups were conducted with 32 public health practitioners who provided public-health oriented services related to mental health promotion and prevention of substance use harms (e.g. harm reduction) in one Canadian province.

**Results:**

Participants engaged in the basic social process of *navigating conflicting value systems*. In this process, they *came to recognize a range of ethically challenging situations* related to health equity within a system that held values in conflict with health equity. The extent to which practitioners recognized, made sense of, and acted on these fundamental challenges was dependent on the degree to which they had developed a critical public health consciousness. Ethically challenging situations had *impacts* for practitioners, most importantly, the experiences of *responding emotionally to ethical issues* and the experience of *living in dissonance* when working to navigate ethical issues related to promoting health equity in their practice within a health system based in biomedical values.

**Conclusions:**

There is an immediate need for practice-oriented tools for recognizing ethical dilemmas and supporting ethical decision making related to health equity in public health practice in the context of mental health promotion and prevention of harms of substance use. An increased focus on understanding public health ethical issues and working collaboratively and reflexively to address the complexity of equity work has the potential to strengthen equity strategies and improve population health.

**Supplementary Information:**

The online version contains supplementary material available at 10.1186/s12889-022-12627-w.

## Background

Promoting health equity and reducing health inequities are considered both moral imperatives and ethical endeavors in public health (PH) [1]. Health inequities are unjust, unfair and result from potentially remediable conditions that impact and are implicated in the development of poor health outcomes [2]. Promoting health equity requires addressing the conditions that produce health inequities through engaging affected communities and taking action on the social determinants of health [1].

Historically, bioethics has been dominated by clinical biomedical issues that are concerned with the relationship between individual providers and clients in the provision of acute care and being able to enact right courses of action [3]. The underpinnings of PH, rooted in social justice, illuminate many ethical tensions in policy and practice related to communities and populations that are not generally identified or of concern in biomedical ethics [4, 5]. To date, there has been almost no attention to and research on the ethical issues inherent in reducing health inequities experienced by PH practitioners as they navigate these issues [6]. For example, there is considerable work related to advocacy for and integration of health equity in various areas of public health practice but little focus on the ethical issues associated with implementation and integration [7–10]. In previous work, we identified and named a range of ethical tensions encountered by public health practitioners working in mental health promotion and prevention of harms of substance use [11]. The purpose of this article is to report a grounded theory study of PH practitioners and their processes of navigating ethical issues related to health equity in PH practice of mental health promotion and prevention of harms of substance use in Canada.

Dominant health care ethics theoretical perspectives and frameworks are primarily attuned to assessing and addressing individual and biomedical issues [12–14]. Several authors have pointed out that ethical concerns in PH have not been adequately addressed through dominant bioethical frameworks [15–18]. Others have noted that PH providers cannot simply adopt the principles of biomedical ethics but require a critical perspective on philosophical and theoretical approaches to dealing with PH ethical concerns [19, 20].

There has been growing interest in and expansion of the field of PH ethics as an area of inquiry separate from clinical bioethics [14, 21, 22]. PH ethics is a field of applied ethics distinct from biomedical ethics in that it: 1) focuses on populations rather than individuals; 2) brings equity to the forefront; 3) considers upstream action on the social determinants of health; and 4) aims to prevent illness and disease [23]. Thus, the ethics of PH is as distinct from traditional bioethics as is the practice of PH from biomedically oriented approaches to care.

From the time of Virchow, known as the founder of the critical public health movement, to the present, PH has placed a strong value on advocacy, attention to inequity, political and structural influences on health, as well as solidarity and interdependence [4, 24]. The driving force of PH ethical frameworks are not only the community and population health-oriented nature of PH [25], but also those of social justice and equity [14]. Thus, the social justice foundations of PH give rise to a different value system than guiding practice in the overall health care system, which despite being considered universal and publicly funded in Canada, continues to be driven by a rationalized illness orientation with associated values of cure, efficiency and cost-effectiveness [26]. In turn, this creates many ethical tensions at the PH policy and program level that arise when working within communities and at the population level [4, 5]. Thus, the location of the PH system in Canada within the larger biomedically-oriented health care system dominated by individualistic values has created value tensions between PH and health care writ large [27].

To advance ethical theory building for PH practice, it is critical to ground ethical perspectives in the everyday ethical concerns that arise for practitioners and decision makers [28]. The goal of this grounded theory study was to describe the basic social process of navigating conflicting value systems, including how PH practitioners identify, make sense of, and respond to ethical issues in their practice related to promoting mental health and preventing harms from substance use. The research questions for this study were: (1) what are the specific ethical issues encountered by PH practitioners in their efforts to reduce health inequities associated with mental health and substance use, and (2) how do PH practitioners navigate and manage these ethical issues in their practice? Our goal is to use insights from this theory in forthcoming work to develop a framework for ethical PH decision making.

## Methods

### Study design

We employed grounded theory methodology for this study because it is useful for exploring, identifying and analyzing complex processes over time, in particular in situations that have not been previously studied or where existing research has left gaps [29, 30]. Specifically, we used Charmaz’ [31] constructivist grounded theory analytic methods. From this perspective, shared understandings of a phenomenon are co-constructed by participants and researchers during the production of and interaction with data throughout the research process.

### Setting and context

This study is one of four interrelated studies in the *Equity Lens in Public Health (ELPH)* program of research [32]. The purpose of this five-year program of research was to guide and inform learning about the integration of an equity lens in PH in the large geographically diverse province of British Columbia, Canada and to contribute knowledge about health inequities reduction during a time of PH renewal in Canada. As part of this renewal, key strategic documents, including *A Framework for Core Functions in Public Health* [33], and the *Guiding Framework for Public Health in British Columbia* [34], identified the importance of applying an equity lens to all PH programs and services. While several Canadian provinces have included attention to vulnerable populations or incorporation of health equity into public health programs, the British Columbia framework specifically names the application of an equity lens as cross cutting theme to be addressed in all public health programs. The *Core Functions Framework* [33] consisted of 21 core programs in four broad areas: health improvement; disease, injury, and illness prevention; environmental health; and health emergency management. In the Guiding Framework, these 21 programs were integrated into seven visionary goals reflecting broad areas of public health focus: healthy living and healthy communities; maternal, child and family health; positive mental health and preventing substance use harms; communicable disease prevention; injury prevention; environmental health; and public health emergency management. A focused provincial mental health and substance use framework was also subsequently developed with the vision of transforming care systems away from acute crisis-oriented approaches toward an equity and prevention orientation across the life course. For this study, in collaboration with regional health authority and government partners, mental health promotion and prevention of substance use harms were selected as the PH areas of focus for this study to learn about ethical decision making in practice.

### Recruitment and participants

In collaboration with health authority partners and through advertising in professional forums, we used purposive and snowball sampling to identify PH practitioners with responsibilities for mental health promotion and/or prevention of harms of substance use in their work. The main inclusion criteria were working in a public health program that had a primary emphasis on mental health promotion or prevention of harms of substance use such as maternal child health programs and harm reduction programs. We aimed to recruit participants who had direct experience with supporting clients in universal or targeted programs intended to reduce health inequities. Participants were invited to contact the research team directly or, through participation in one of the three other ELPH studies, participants were asked if they would be willing to share their contact information to learn more about this study.

There were 32 participants in this study (28 female and 4 male), from all geographical regions in the province (*Fraser Health, Island Health, Vancouver Costal Health, Northern Health, and Interior Health*) as well as the BC Provincial Health Services Authority, which plans and coordinates access to specialized health-care services including mental health and substance use services. Participants held PH roles within harm reduction programs, pre and postnatal support, and HIV and communicable disease programs. These programs were funded and primarily delivered through health authorities which are quasi governmental regional bodies responsible for all of the health care services in the province including public health.

Eleven participants self-identified as working in traditional PH staff roles (e.g. PH workers or nurses) and 21 worked more explicitly in harm reduction and sexually transmitted infection programs. Twenty-five of the participants (78%) identified as Registered Nurses. Overall participants had practiced for an average of 6.05 years (range 6 months to 20 years) in their current position and an average of 10.26 years (range 6 months to 40 years) in PH. All but one participant had completed post-secondary education programs. These demographics reflect the age, gender, and educational characteristics of the general Canadian nursing workforce [35]

### Data collection and analysis

We conducted 29 semi-structured individual interviews (primarily via telephone) and one face to face focus group with three participants between November 2014 and February 2016. Interviews lasted approximately 60 minutes. Semi-structured and open-ended interview questions were used to encourage responses important to the participants. We recorded and transcribed interviews verbatim, and transcripts were verified by the study coordinator. Following each interview, observations and reflections were recorded and integrated into the analysis. Data were analyzed using NVivo© Version 10.

Five of the six study team members (four faculty and one research assistant) read the interviews and generated a set of inductive codes for the coding framework from the first few interviews. The research assistant continued coding the interviews line-by-line. Participant words were retained when appropriate in code names. Analytic distinctions were established through the inductive process of constant comparison, an integral part of the grounded theory analytic process that helps researchers move codes into higher level concepts while staying close to the data. Specifically, we compared incidents within the same interview and compared incidents and statements across interviews. Memoing and diagramming documented the analytic process and supported continued conceptualizing and theorizing. We held in-depth team discussions to further the data analysis through establishing relationships and differences between concepts, ultimately developing a grounded theory [31].

### Rigor in the study

We took detailed notes during data analysis to create an audit trail of our analytic process. The research team used reflexivity to maintain awareness of structural influences and power relations throughout the research process. The sixth member of the team held the role of knowledge user and reviewed the grounded theory and the manuscript and provided feedback on credibility, originality, resonance, and usefulness, hallmarks of quality for constructivist grounded theory [31].

## Results

**Fig. 1:**
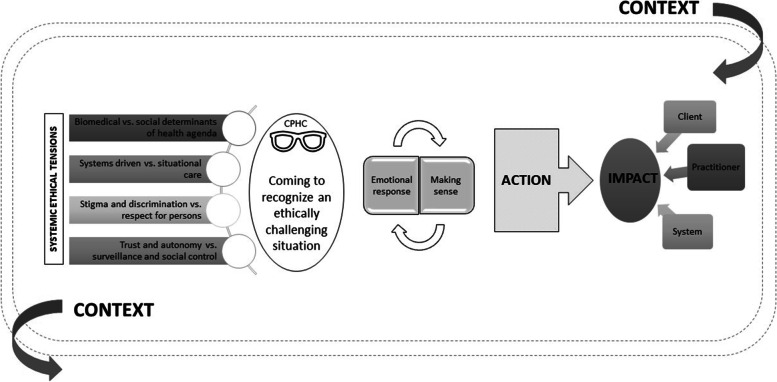
The process of health equity work in public health: Navigating conflicting value systems

Grounded theorists assume that participants in the study share a basic social problem with which they are grappling. In this situation, the basic social problem that participants experienced in their health equity work was a set of ethical challenges that arose from the disjuncture they experienced between the values and goals of the healthcare system (including the PH system) and the values, goals, and standards that guided their practice. This problem was then resolved or managed by the participants through engagement in some type of action named as either a basic social process, a social-psychological process, or a social-structural process [29]. In this study, to address this basic social problem, participants engaged in the basic social process of *navigating conflicting value systems* in their health equity work in public health (Fig. 1).

Participants identified *coming to recognize a range of ethically challenging situations* related to health equity within a system that held values that conflicted with their own. The extent to which an individual practitioner recognized these ethical challenges, however, was dependent on the *degree to which they held or embraced a critical PH consciousness.* Clearly, the recognition of ethical issues informed the way that they *responded emotionally to, made sense of* and *took action on* these issues. These ethically challenging situations, regardless of the extent to which the practitioner held a critical PH consciousness, *impacted* clients, practitioners and the health system itself through this process. Most significantly for PH practitioners, they experienced *dissonance* when working to navigate ethical issues related to health equity in their practice in a health system with different values.

### Coming to recognize ethically challenging situations

Participants described a range of ethically challenging situations that centered on differing agendas between provincial or regional health systems and PH practice. These were situations in which they experienced value conflicts between system or organizational values and the values of PH. Participants described ethically challenging situations as those that arose when PH values or ethical principles were overlooked, dismissed, eroded or violated. These situations reflected tensions between competing health system and health equity values.

We briefly introduce four core ethical tensions here and describe these in more depth elsewhere [11]. The tensions were: 1) biomedical versus social determinants of health agenda, 2) systems driven care versus situational care, 3) systemic stigma and discrimination versus respect for persons, and 4) trust and autonomy versus surveillance and control. To varying extents, participants recognized that they were attempting to do health equity work “on the ground” in organizations in which the dominant health system focus was biomedicine with lack of value for PH generally or health equity specifically. They also highlighted how the pressure of meeting systems needs and requirements (manifested by practice structures such as procedures, guidelines, checklists) drove PH work rather than the situational needs of clients. Systemic stigma and discrimination were identified as pervasive barriers within the health care systems, particularly in relation to mental health and substance use, and participants voiced concerns about sending clients to services knowing or not knowing how they would be treated. Lastly, participants identified that they often experienced ethical tensions in situations in which preserving trust and choice came into conflict with requirements for surveillance and responses that emphasized social control rather than social support. They reported having to navigate carefully the issues of confidentiality to ensure public protection and to preserve trust and autonomy.

The extent to which public health providers recognized these tensions as ethical challenges was dependent on the degree to which they held or embraced strong commitments to social justice and health equity, which we have described as a critical PH consciousness. Participants described formative experiences, including how they were parented, their age and involvement in other societal movements, and their exposure to people who created a milieu that contributed to their awareness of social justice issues. One participant described their own process of developing social awareness:

*I think as I got into nursing I met – inside and outside of nursing – you know, going to college and university – I met women that were involved in the women’s movement. I shared a place with a group of other women who were very socially active...we were all pretty activist in our hearts…then it’s like a snowball around social justice issues. (S4*-*31)*

At that time, she was supported by a progressive manager who encouraged creativity and did not micromanage; thus, she felt an increased sense of autonomy and confidence in speaking out. However, this participant also shared that that their equity perspectives were quite different from many of their peers who were raised in more conservative environments and had more conservative experiences.

The depth of reflection on the situations that participants described was moving, and even motivating at times. In their personal reflections, they described processing these emotional responses as part of how they came to make sense of the situation.

### Responding emotionally

Participants described experiencing a range of emotional responses during and after recognizing an ethically challenging situation. In some cases, it took time for them to reflect on what had happened and link what they were feeling to an ethical tension or moral distress or uncertainty. Moral distress occurs when a practitioner knows the right thing to do but is unable to enact an ethical decision due to internal or external constraints [36, 37]. This is in contrast to moral uncertainty in which a practitioner may not be aware of the right actions. Primarily participants expressed negative self-oriented emotions, including feeling uncomfortable, uneasy, uncertain, sad and miserable. They worried whether they were doing the right thing and felt personally responsible for the outcomes even though, for the most part, what happened was out of their control.

Well unfortunately with the client, I’m not sure, the client is now missing and I think that the client is probably returned to an exploitative situation because that was the only option. I honestly have to say I probably cried, that happened on a Friday…. I did everything I could, and I just couldn’t, you know. We determined that there was no solution and I probably cried for just hours that weekend, you know. (S4-28)

In addition to self-oriented emotions, participants also identified emotional responses directed at the system. They feared that the equity perspective that would have facilitated access to appropriate resources was losing ground to the values of the dominant biomedical system. This self-reflection not only facilitated identification of emotional responses, but it also initiated a process of thinking through and making sense of the different elements of the ethical situation, including contributing factors, context, and personal perspectives.

### Making sense

Making sense refers to the process of how PH practitioners thought and reasoned about the various ethical situations that they encountered. These interpretations then informed the actions that they took or did not take. Participants described three strategies that they employed in making sense: 1) destigmatizing their perceptions, 2) learning to think ethically together; and 3) talking through situations collectively.

#### Destigmatizing their perceptions

Many participants experienced a steep learning curve when they first started working with populations experiencing systemic and structural vulnerability. Their initial perceptions were often biased and judgmental, based on stereotypes and past personal and practice experiences. Participants described the processes that they used to acknowledge and examine these biases, including framing the issues differently (such as considering the social determinants of health), reflecting on why they felt uncomfortable, and seeking out opportunities to learn from other professionals and those with lived experience. A participant shared the personal story of growth:

I was very judgmental, had a lot of stigma … over the years that was unlocked for me and I forced myself to be a street nurse because I thought “you know [name]… you have way too much stigma and discrimination towards this bunch of people.” So that’s what I did. I forced myself to go into this work to change myself. And over time it did happen, and I began to understand. And I think that’s the crux, and it was for me. And from talking to the students, that they’ve had no exposure to date, they don’t understand, right? And once they hear people’s stories out there, I tell you, they come back with such a different perspective. Such a different perspective. It’s unbelievable. (S4-20)

This participant, a street nurse with their practice focused on supporting marginalized populations characterized by extreme poverty and homelessness, highlights a change in thinking over time, increasing their critical PH consciousness and capacity to think more critically about their practice, through intentional learning facilitated by continual engagement with clients. An important aspect of this process was recognizing the history and context in which an individual, family or community is embedded. When they framed this bigger picture for themselves, they gained insight into the broader context of the situations that they encountered helping them navigate through the issues and identify appropriate actions. Other participants with a more underdeveloped sense of PH, for example those newer to PH, had comments that were more biomedically focused. For example, one participant shared that within their team, understandings of harm reduction varied greatly:

*I’ve had colleagues say, “Oh, I had a great harm reduction conversation with this client about cocaine. I told them never to use cocaine, and how bad it is, and how it will mess up their nose, and what their future will be like. (S4-15)*

This colleague demonstrated a narrower, biomedically-focused approach to harm reduction, thus providing less opportunity to explore equity-oriented solutions to addressing the harms of substance use.

#### Learning to think ethically together

Some participants noted that they used or wanted to learn to use the language of ethics to make sense of and bring ethical issues to light. They shared that they often did not use ethical language in conversations with their colleagues but in hindsight saw that they were dealing with ethical concerns:

*I always feel I fall short a little bit in these conversations because I think sometimes for me that the ethical uncertainty and ethical distress, they’re terms that I think I experience all the time but it’s hard to language. (S4-08)*

They reflected that it would be helpful to be able to articulate what the ethical issues are and how to address them in ways that were not just mechanistic, but were strongly grounded in health equity principles.

Participants also identified the importance of being able to identify core ethical values in their workplace, including health equity and social justice, and infusing these values through their decision making and actions.

I think that… it’s a collective goal of our staff to increase equity to healthcare and better wellbeing for the folks that we work with. I think that we’re practicing in alignment with our values as much as we can. As a supervisor, helping staff to be in as much in alignment with their values is one of the most important things and I see it as vital to staff retention and staff health and their ability to support other people. (S4-08)

Being able to practice in teams in which these shared values were held was seen to promote work satisfaction and retention, maximize the quality of care, and avoid staff burnout. Some participants wondered if PH and street nursing attracted a “certain kind of person” that shared these ethical and political values. Some organizations had more control than others over how to hire people specifically for these expressed interests and commitments, with a greater challenge noted in collective agreement environments.

#### Talking through situations collectively

The support of like-minded colleagues was noted as being critical for being able to talk through issues, explore options for action and confirm decisions. This support may be formal (e.g., through established communities of practice, professional organizations, reflective supervision, or team debriefing) or informal (e.g., friends who are nurses but not colleagues).

We don’t work in isolation, that’s for sure. We do have colleagues and people that we can talk to within our team, run things by. You know, “what do you think I should do in this case?” And I’ve often heard through talking with colleagues, we’ve often asked that question: “okay, how far do you go with this?” or “well do you really think that’s a good idea?” So those kinds of phrases that let me know that support that I have from my colleagues is excellent. And I really trust their opinion, and I know I’m not alone in doing this work, right? (S4-12)

Alternatively, practitioners lost a significant source of support and education when workplaces and teams were structured in such a way that there was no time or opportunity to consult and engage in dialogue. There was also reduced trust within the team when there was continual change in team composition, membership and leadership, in particular if colleagues were not seen as practicing from the same paradigm:

*Not everybody is sympathetic. They can’t understand why they are doing the things they are doing. Yeah, ethically that is another challenge as well, dealing with your peers and dealing with fellow healthcare nurses that are not on the same page (S4-20).*

Some participants shared that they had to make decisions about whether it was worth their time or energy to have conversations with or challenge the understanding of certain colleagues. They then could, in fact, feel isolated and in some cases even stigmatized within their teams. Overall, the outcome of this sense making process, whether internal or collective, was for PH practitioners to then feel more confident in making decisions on what actions were appropriate to take to address the situation.

### Taking action to address an ethical tension

Taking action refers to the approaches and strategies that PH practitioners used to respond to and resolve the ethical situations they encountered in their practice. Once they recognized and made sense of an ethical situation involving health equity and reflected on and processed their emotional responses, participants acted based on their level of critical PH consciousness and the extent to which they were in a supportive practice environment often having to navigate conflicting value systems. This included: 1) advocating and 2) doing whatever it takes.

#### Advocating

The concept of advocacy was framed by participants from systems responsibility and population rights perspectives. Health systems were described as operating from standardized efficiency approaches (i.e., ticking the boxes on a form and saying the job is done) in which there was no place for caring, creativity and individualizing. Advocacy strategies included getting on committees to move issues forward, lobbying for additional resources, channeling venting into action, and keeping an eye open for timely opportunities to insert key equity messages.

I think what we really need to do as healthcare workers, as women, and as people in these jobs, is we need to have, have a bigger political voice. Because nothing’s going to change on the lower levels unless we’re more vocal about it. So for me, it’s hard because I don’t like to speak out to the “up-aboves” and I don’t like to push. But unless we, as groups that have this, or you know, as being a mom myself, for getting people organized together, voting and going to more political action, I don’t think that it’s going to change. (S4-04)

Advocacy was often focused on creating opportunities for the voices of people with lived experiences to be heard and valued at decision-making tables, including creating and resourcing peer advisory committees. One participant described how they integrated client voice into ongoing strategic community planning:

We are about to go into a round table process with [community leaders] to talk about moving forward on establishing supervised consumption services and what that could look like. And a big part of the campaign is we have a peer advisory committee, so we have people with experiential knowledge related to illicit drug use who are involved with the campaign and who we want to foreground their experience and their voices in moving forward on this stuff. You know, the whole “nothing about us without us” concept. (S4-08)

This type of advocacy for experiential voices that are often excluded to be included is consistent with key strategies for promoting health equity through client and community engagement and social inclusion [11].

#### Doing whatever it takes

Participants demonstrated through their stories that they were persistent, resilient, creative and sometimes subversive to obtain the resources and supports needed by their clients in spite of conflicting value systems. For example, some participants leveraged their relationships with other programs and agencies and bartered resources. They circumvented organizational policy and role definition barriers. “Work arounds” were related to accessing resources, referring quickly, sharing information about clients across systems, and maximizing clients’ ability to meeting criteria for accessing services such as housing.

I have other methods that I can obtain things, you know. And it’s not illegal just so you know! It’s just people I can call and say “can you send this?” and they do, and we trade things back and forth, or I can go up to the local hospital and say “look, this is what I need. It’s a lot less expensive for me to give 10 of these than it is if we’d have to send the family in through emergency.” So that’s not a threat. When you explain it to somebody like that, they’re like “okay what do you need? (S4-16)

Some participants found themselves in ethically challenging situations in which they either had bend the rules to create trust or subject their clients to greater social control. In this example, the participant chose to work the system rather than disadvantaging the client who may not persist further after experiencing a barrier:

And I’m not your average nurse (laughter). You know, I kind of bend the rules for clients where I can, and I think they really appreciate that. And I think it’s just kind of, you know, whether that’s sending in an STI sample that’s for somebody without a PHN [Personal Health Number] or whatnot. They just really appreciate that kind of thing, and it goes that extra mile. And then you build trust with them, and then they tell their friends about it and all of a sudden you’ve got a bigger marginalized clientele that’s coming in and people start to know you for being a more relaxed nurse. (S4-26)

Taking action was not achieved in discrete actions that were easily laid out in a stepwise process. There were multiple actions and approaches based on how participants made sense of ethically challenging situations that were complex and intertwined in order to navigate conflicting value systems.

### Living in dissonance: The impact of navigating conflicting value systems

When PH practitioners recognized and attempted to navigate ethically challenging situations there was an impact or effect on them, and also on their clients and the health system in some way. The result of the process of navigating conflicting value systems was experienced as dissonance by participants, where they knew what was possible in a strong PH system focused on health equity, yet they saw the reality of the bio-medically oriented system in which health inequities and marginalization were experienced by their clients. PH practitioners thus often felt: 1) powerless as they witnessed inequities; 2) feeling like a stranger in a strange land; and 3) like they were living in a middle ground. Ultimately, they felt caught between their employer and their professional obligations and the feeling of responsibility, which ran the risk of leading to burnout.

#### Witnessing inequities

Participants frequently witnessed and heard stories of health inequities lived by the clients they served and yet had no power or resources to address these inequities. PH practitioners described seeing limited to no systemic response to addressing the determinants of health in a substantive way. It weighed heavily on them when they saw what needed to happen but were not able to do what they knew could make a difference. That, coupled with high workloads, was felt to lead to poorer personal outcomes for participants who experienced burnout.

Well I think that’s… certainly part of the burnout is you go home from work for the day and you think “oh, I didn’t get that done, I didn’t get that done, I haven’t been to that school for 2 weeks, and I need to…” you know? You carry it home with you. As hard as you try not to, you know I wake up in the night thinking about this kid or that kid or that teacher and it takes a lot of energy to put that aside and just be at home. You know, “that’s my job” it’s separated out, compartmentalize. That takes energy. (S4-18)

Participants described feeling a sense of powerlessness in holding the position of continually bearing witness to inequities experienced as mentally, emotionally, and physically exhausting. Compassion fatigue and burnout were readily evident in participant stories.

#### Feeling like a stranger in a strange land

Depending on the work team and organization, some practitioners felt as if they were standing alone, isolated in their ethical and moral distress, in their work to reduce health inequities. They felt responsible for taking action and being the voice of advocacy on their team. One strategy used by practitioners who felt isolated in their approach to their work was to find other like-minded people external to their organization with whom they could work through ethically challenging situations. For example, one participant noted:

*Well it really depends, because I have a little gaggle of friends who are nurses, and some are not, but mostly nurses who I can talk to about this and they agree and I don’t feel like I’m a stranger in a strange land*. *(S4-02)*

Another practitioner also identified the feeling of isolation on the team stating: *“But it is quite embarrassing to always be the person bringing it up. Like it’s like beating the dead horse.,,So it’s awkward. But it’s not something that’s okay to just see what happens and ignore it” (S4-15).* The impact of being alone or being embarrassed affected the way they reflected on their quality of work life and ability to do their job and thus their willingness to stay in their position.

#### Living in the middle ground

The final impact of navigating conflicting value systems was to make the decision to settle on something less than their ideal. One participant framed their tactic as resigning to live in the middle ground, having considered the benefits and drawbacks to continuing to practice based on PH values.

I personally I think I’ve gotten to a point in my life where I feel like I’ve sort of made a deal and I live in the middle ground, meaning there are certain limitations to my work that I have to accept if I’m going to work for this organization, which there’s lots of perks, including a nice stable pay cheque, whereas if I quit and work for [another community organization] for example, where you get to be a bit more radical, some of those perks are gone. So, I feel like it’s trying to balance personal responsibilities for my family and my personal integrity and then trying to find a way within [health authority], which is a very hierarchical bureaucracy – and I mean I feel like I work in a good little pocket where we try and subvert some of the policies. (S4-07)

Working out this balance was important for PH practitioners as they came to understand how to survive in conflicting value systems and still maintain enough of a degree of health equity thinking that they could feel they were meeting their ethical obligations. Ultimately, some participants left the workplace when they felt that they were no longer able to make sense of or live ethically with the work that they were doing.

## Discussion

Overall, our findings support research emerging from the evolving field of PH ethics. Specifically, the findings highlight the tension that arises for PH practitioners when the obligation to focus on the health and well-being of populations is situated within health care delivery systems that are primarily structured to focus on individuals and acute care needs [14, 38]. The basic social process identified by PH practitioners in this grounded theory study was navigating conflicting value systems to promote health equity in public health. The process of navigating these tensions began with recognition that there was an ethical issue at play. This awareness, and the sense making and action that followed, was contingent on the degree to which an individual had developed critical PH consciousness. The recognition of ethical issues in PH practice spanned the dominant health system values of a biomedical agenda, systems driven care, systemic stigma and discrimination, and surveillance and control. These values did not align with those of the PH practitioner, which included health equity, situational care, respect for persons, and choice and relational autonomy [4]. Practitioners often had an emotional response to this misalignment, frequently interpreted as an ethical tension or a sense of moral distress. They made sense of the ethical issue through internal and collective reasoning processes, and shifted to action including advocacy through engaging with the system and encouraging participation of people experiencing inequities. The overall impact of navigating conflicting value systems was one of living in dissonance, often caught between what they knew they ought to be doing and what they were expected to do.

The moral foundation of PH practice has been described as determining the balance between what PH is and what PH practitioners think it should be [39]. In this study, participants demonstrated their moral foundation and ethical awareness through language that aligned with the concept of critical PH consciousness (CPHC), based on Freire’s [40, 41] framework of critical consciousness. Freire [40] posited that learners who connect with their personal, intellectual, and emotional experiences can discuss them with others, resulting in a reflective reading of the world (or conscientization), concentrating on changing societally embedded inequities. Participants in this study varied in the extent to which they demonstrated CPHC. Some PH practitioners expressed a high degree of CPHC, citing a full range of ethical issues encountered in doing health equity work spanning multiple contexts. The depth of reflection on these situations was moving and motivating. Their personal reflections included the identification and processing of emotional responses which contributed to making sense of the situation. Those demonstrating a high degree of CPHC were relentless advocates and did whatever they could to meet the client’s needs, even when it meant circumventing rules and system limitations. Other PH practitioners were less aware of the broad range of ethical issues related to health equity in their work, thus expressing less of a CPHC and holding a more restricted idea of what they viewed as an ethical issue. The degree to which participants held a CPHC was integral to the kind of impacts they identified clients as experiencing, as well as impacts on themselves and others. It also subsequently expanded or limited the possibilities for action that they considered. However, a high degree of CPHC was noted to make them more vulnerable to experiencing moral distress and thus burnout.

PH practitioners’ conceptions of ‘doing good’ did not come solely from abstraction or theory. It appeared that their obligation to act arose from their situated position proximal to service users’ lived experience. We noted that participants with limited direct exposure to lived experiences of marginalization and less awareness of and commitment to equity and social justice were less likely to see inequities as structural and more likely to locate responsibility for inequities within the individual and their behaviour or lifestyle. PH practitioners that had a critical socio-structural understanding of health issues were more likely to have had experience working with marginalized populations or in some cases had their own lived experience of or identification with issues including substance use and mental health. Thus, the capacity to develop CPHC seemed to have proximity, dose, and exposure dimensions.

Practitioners routinely experienced ethical concerns resulting from their daily encounters with clients, yet had few organizational resources available to support their ethical decision making. While there may be ethics consultation support in healthcare organizations, this support was often geared toward individual and biomedical issues with few supports for PH practitioners grappling with everyday ethics in their practice. Ethical framework resources are needed and may be useful to guide reflection in practice. However, traditional biomedical frameworks will not capture such tensions around health equity. New frameworks are needed that specifically reflect PH values. Lee [38] suggests that even if the PH ethics field has not yet come to a place of agreement about a unifying theory, framework, or set of principles, there is still an immediate need for practice-oriented tools for recognizing ethical dilemmas and supporting consistent and defensible ethical decision making.

Providing time for critical reflection and dialogue is another essential resource in practice and in education to support PH practitioners to develop their CPHC, process their emotional responses, and problem solve ethical challenges. Ethical dialogue with a supportive practice team could serve to both decrease moral distress and facilitate development of CPHC. Hamric and Wocial [42] describes the creation of moral spaces and development of moral communities to facilitate interprofessional dialogue. Combined with decision making tools or frameworks, this reflection and dialogue can serve to not only alleviate moral distress, but also create avenues for collective social justice action.

Ortmann et al. [43] suggest that because public health itself is practical, pragmatic, and community oriented, ethical frameworks to guide public health practice must then be culturally, socially and politically aligned, and grounded in the public health values of health equity and social justice. However, the commitment to health equity and social justice has become difficult to sustain for many Canadian PH practitioners over the last two decades as health systems have shifted to corporate models of service delivery and as practice has increasingly become controlled [44–46]. It appears that in the face of these challenges, PH practitioners felt they had less support for responding to inequities, meeting the unique needs of individuals, or advocating for systemic concerns. Thus, when participants recognized and made sense of ethical issues and then took action on health inequities, they were demonstrating their own professional commitments to social justice rather than enacting organizational commitments to health equity. In other work, we have identified that health equity is often a value in name only and one held by individual practitioners and leaders but not necessarily organizations [47].

Some participants suggested that the current health and political environment in general is hostile to working toward social change, resulting in some PH practitioners going underground in the form of finding ways to work around the system to attempt advocacy and achieve what they feel is best for the client. “Workarounds”, known as strategies that differ from prescribed procedures taken to temporarily fix or circumvent problems, develop when the conditions and pressures of work in complex systems meet the structural constraints of these systems [48]. Berlinger has characterized workarounds as ethically significant as they emerge from the tension that arises when complying with rules is impossible to reconcile with the demands of work [49]. From a complex systems perspective, they reveal the incommensurability between professional values and the demands of health care organizations as workplaces under pressure [49]. Although advocacy is clearly identified as a PH competency it was felt to be often negated or not encouraged in health care systems, even in PH systems that philosophically should be oriented this way [50, 51]. Cohen and Marshall [52], in their scoping review of public health advocacy, found that the literature reflected a neoliberal preoccupation with individual responsibilities for health, thus reproducing rather than resisting corporate politics.

In addition to advocacy, the *Core Competencies for Public Health Practice in Canada* [51] are grounded in the fundamental values of health equity and social justice with an assumption that all health professionals share and can operationalize these values, despite differences in identity, social location, and cultural background among practitioners themselves. Furthermore, PH practitioners who are more engaged with traditional epidemiology and virology, may embrace biomedical ethics more than those whose work is in social epidemiology [53]. Alternatively, PH practitioners have clear real-life commitments to social justice and health equity, and may therefore have had greater opportunity to develop CPHC and have the capabilities for enacting or *doing* social change.

Overall, the process of navigating conflicting value systems required PH practitioners to have a high level of ethical awareness to understand the systemic challenges of complex healthcare delivery, as well as address the societal challenges of stigma and discrimination that accompanied mental health and substance use concerns. In this study, participants articulated the distress and high emotional costs that came with this awareness. Similar to other research on moral distress in health, they expressed frustration and powerlessness [54, 55]. Unintended consequences for the PH practitioner of not resolving these tensions may include disenfranchisement, disengagement, attrition, decreased quality of care, and reduced population health impacts [54, 55].

The participants in this study, whose practice focused on promoting mental health and reducing the harms of substance use, were for the most part clearly committed to equity and social justice and had developed the ability to vision and effect social change in a strategic way. However, they did not appear to draw on a shared language or coherent theory or framework to identify, make sense of and act on the ethically challenging situations they faced in their work. Additionally, the organizations and systems within which they worked were usually not designed or resourced to support the work of social change. Beyond inclusion of concepts such as advocacy in core competency frameworks, explicit ethical guidance for PH, in particular at the direct care level, is lacking [50, 51, 56–58]. The resources that are available tend to be framed around the biomedical concepts of autonomy, beneficence, non-maleficence, and justice [17]. Although health equity and social justice are core values of public health, there is not yet consensus on a practice-oriented ethical framework that integrates these differences into guidance for decision-making guidance [59].

Health equity work is not an abstraction for PH practitioners as they have acquired experiential knowledge about the messiness, challenges, and successes of this work, especially in the fields of mental health and substance use. For example, although there is a wealth of research that has exposed the systemic stigma in acute health care settings and the ways in which health care providers are complicit in reinforcing stigma, there is still little evidence available on how PH practitioners navigate systemic stigma [60, 61]. An increased focus on understanding the ethical issues and working collaboratively and reflexively to address the complexity of equity work has the potential to generate new solutions and/or strengthen equity strategies.

## Strengths and Limitations

A key strength of this study was that participant experiences reflected a wide range of organizational and geographical contexts across the Canadian province of British Columbia. Conversely, British Columbia represents one specific provincial political and economic healthcare jurisdictional perspective, so findings will need careful consideration when being translated to different contexts. Ethical issues were limited to those identified related to mental health promotion and prevention of harms of substance use practice. While these are two practice areas generate substantial ethical concerns in part due to the stigma and discrimination associated with these issues, it is important to recognize that ethical issues and trade offs will manifest differently in different areas of public health. Furthermore, these findings reflect the experiences of a mostly female, mental health and substance use-oriented public health workforce. While this grounded theory may hold promise for application across the many functions of PH, it is unlikely that any one PH ethics framework will be applicable across all PH ethical challenges. We recommend future research to study the applicability of this grounded theory across the diverse PH core functions and the specialized practitioners associated with these functions.

## Conclusions

Enacting PH values is vital when working in areas where reducing health inequities is the goal. Lee and Zarowsky [39] suggest that because these values differ from those of biomedicine, the processes and tools that are needed to make ethically-supported decisions also need to differ. We have aimed to contribute to the understanding of how PH practitioners, through promotion of and commitment to health equity, negotiate and advocate within health and social systems to assist clients in meeting their needs related to the social determinants as well as advocate for inclusion of their voice within health systems. Kass [14] states that “public health ethics is, ultimately, a practical field” (p. 239). The PH practitioner who values collectivism and solidarity, and is working toward social justice, is one who is more likely to experience ethical tensions and dissonance in their practice. They may benefit from an ethical framework that can guide them in their everyday practice, firmly grounded in the core values of PH.

This study also contributes to knowledge of how health care providers in PH are finding ways to empower themselves. However, while health systems may purport to hold goals of health equity, valuing health equity, and taking action to promote it remain challenging [62]. These findings point to the need for organizational strategies and supports that are attentive to listening and seeing moral distress as an opportunity for systems reform specifically related to promoting health equity in organizations. Health equity is, after all, a stated goal of health systems as well as public health [63].

## Supplementary Information

**Additional file 1.**

## Data Availability

The datasets generated and/or analysed during the current study are not publicly available due to the sensitive and confidential nature of the data.

## Abbreviations

CPHC: Critical public health consciousness
PH: Public health
