# Supplementary material for: Navigating conflicting value systems: a grounded theory of the process of public health equity work in the context of mental health promotion and prevention of harms of substance use
Source: BMC Public Health. 2022 Feb 1;22:210. doi: 10.1186/s12889-022-12627-w (PMC8805448; doi:10.1186/s12889-022-12627-w)
Supplement: Supplementary file 1 — Additional file 1. [file 12889_2022_12627_MOESM1_ESM.docx]

Equity Lens in Public Health

Interview Questions – Public Health Ethics Research

Please note that we each interview is different and we may ask different questions to explore other topics that come up, much like a conversation.

1. Tell me about your work.
2. What activities are you involved in related to mental health promotion?
3. What activities are you involved in related to preventing the harms of substance use?
4. What activities are you involved in related to prevention of mental illness or mental disorders?
5. Can you tell me about health equity in your work?
6. We’re interested in situations or examples in which you have experienced ethical issues or uncertainty in your work related to health equity and MHSU. Ethical uncertainty occurs when one feels indecision or a lack of clarity, or is unable to even know what the moral problem is, while at the same time feeling uneasy or uncomfortable. What are some examples of ethical uncertainty that you encounter in your work? This uncertainty may be negative or positive, large or small.
   1. Describe the situation
      1. Where were you working when it happened?
      2. What were your thoughts and feelings when it happened?
      3. When did it happen?
      4. How often does or has this happened?
   2. Who was involved?
   3. Were you able to work through or resolve the issue? If so?
      1. What was your decision making process in determining whether or not to take action?
      2. How did others respond?
      3. What were the outcomes of your action, if any? (E.g. organizational policies, different practices?)
7. How did this experience impact you?
   1. What have been your reflections, if any, on this experience?
   2. In retrospect, is there anything you would you have done differently?
   3. What was the impact at the time?
   4. Have there been any other impacts since the experience?
   5. How did the experience impact others involved in the situation?
   6. How was the issue resolved?
      1. Did it compromise your ethics? In what way?
8. What support or resources, if any, did you have available to you to help in addressing the ethical uncertainty described above?
   1. What ethics resources, if any, did you access?
      1. How did you use these and were they helpful?
   2. What other ethics resources are you aware of in your organization?
   3. Did you access any ethics resources outside of your organization?
      1. How did you use these and were they helpful?
   4. Of the resources you accessed, what did you find helpful?
      1. What was not helpful?
9. Is there anything else you would like to add?

## Demographic Questions

Age:

Gender:

Educational Preparation: *(diploma, degrees, and year received)*

Years of experience in Public Health:

Current Job Title:

Current Job Responsibilities:

Length of Time in the Current Position:
